# Vibrator Massage of the Uterus

**Published:** 1897-04-24

**Authors:** 


### VIBRATORY MASSAGE OF THE UTERUS.

In cases of hæmorrhage due to atony of the uterus Dr. Kumpf, of Vienna, recommends subjecting that organ to vibratory massage. The accoucheur stands on the right side of the patient and applies the tips of the fingers of his right hand on the abdomen, perpendicularly to its surface, at a point about midway between the umbilicus and the symphysis pubis. He then pushes back the abdominal wall towards the vertebral column, performing at the same time as rapidly as possible a series of vibratory movements with the whole arm kept rigid. This manœuvre is said to have on the uterus a much more powerfully stimulating action than simple friction, however energetically applied. The outlines of the uterus soon become distinct. The surgeon then changes his place and exerts the same mode of massage on the posterior surface of the uterus, which is now pressed against the symphysis pubis. In about ten seconds the uterus is in a good state of contraction. Such, at any rate, was the result in two (!) cases* in which Dr. Kumpf made use of his method.

* Medical Week.

